# Fiber-optic annular detector array for large depth of field photoacoustic macroscopy

**DOI:** 10.1016/j.pacs.2017.01.001

**Published:** 2017-01-25

**Authors:** Johannes Bauer-Marschallinger, Astrid Höllinger, Bernhard Jakoby, Peter Burgholzer, Thomas Berer

**Affiliations:** a Research Center for Non-Destructive Testing GmbH (RECENDT), Altenberger Straße 69, 4040 Linz, Austria; b Institute for Microelectronics and Microsensors, Johannes Kepler University, Altenberger Straße 69, 4040 Linz, Austria

**Keywords:** Photoacoustic imaging, Photoacoustic macroscopy, Interferometry, Fiber-optics imaging, Optical ultrasound detection

## Abstract

We report on a novel imaging system for large depth of field photoacoustic scanning macroscopy. Instead of commonly used piezoelectric transducers, fiber-optic based ultrasound detection is applied. The optical fibers are shaped into rings and mainly receive ultrasonic signals stemming from the ring symmetry axes. Four concentric fiber-optic rings with varying diameters are used in order to increase the image quality. Imaging artifacts, originating from the off-axis sensitivity of the rings, are reduced by coherence weighting. We discuss the working principle of the system and present experimental results on tissue mimicking phantoms. The lateral resolution is estimated to be below 200 μm at a depth of 1.5 cm and below 230 μm at a depth of 4.5 cm. The minimum detectable pressure is in the order of 3 Pa. The introduced method has the potential to provide larger imaging depths than acoustic resolution photoacoustic microscopy and an imaging resolution similar to that of photoacoustic computed tomography.

## 1 Introduction

Photoacoustic imaging is capable of acquiring images at scales ranging from resolved sub-micrometer features in shallow depths to whole body scans of small animals with a resolution typically in the order of hundred micrometer [1]. The huge size range is covered by different implementations, where, as a rule of thumb, the achievable spatial resolution is typically around 1/200 of the imaging depth [2]. In acoustic-resolution photoacoustic microscopy (AR-PAM) a focused piezoelectric ultrasound transducer is used to acquire the generated ultrasonic waves along the axial direction, i.e. an A-scan [3], [4], [5]. By raster-scanning, individual A-scans can be composed to form a two-dimensional or three-dimensional image. In AR-PAM the best achievable axial resolution is mainly defined by the bandwidth of the ultrasound detection. Depending on the imaging depth, the frequency-dependent acoustic attenuation within the sample may, however, reduce this resolution. The lateral resolution is additionally limited by the acoustic numerical aperture (NA) of the transducer. Here the region of high spatial resolution is obtained only within the Rayleigh-length and the lateral resolution degrades outside of this region. As a result on the dependence on the acoustic NA, the element size of the transducer has to be increased for large imaging depths, in order to maintain high spatial resolution. However, large-area high-frequency focused piezoelectric transducers are bulky, challenging to fabricate, and hinder sample illumination. Thus, the imaging depth in AR-PAM is usually restricted to a couple of millimeters [6]. In order to speed up data acquisition and to increase imaging depth, in photoacoustic computed tomography (PACT) transducer arrays are used instead of scanning a single element transducer [7], [8], [9], [10], [11], [12]. The lateral resolution is then given by the acoustic NA provided by the ultrasonic array detector. If the ultrasonic transducer covers a large acoustic NA, e.g. by using a cylindrical configuration or by scanning the array around the specimen, good lateral resolution can be achieved also in relatively large depths. However, in many practical applications the specimen can only be assessed from one side, thereby limiting the acoustic NA and the resulting lateral resolution. In PACT, commonly one-dimensional detector arrays are used [8], [9]. Here the acoustic NA is different in the active and passive planes. The active NA is given by the total array length and is typically much larger than the passive NA, which is limited by the width of the array. Thus, the lateral resolution differs between the active and passive planes, and high-resolution can only be achieved in the active plane. In our approach, photoacoustic scanning macroscopy (PASMac), we try to bridge the gap between AR-PAM and PACT, by providing imaging depth and resolution as typically achieved in PACT, but obtained with a scanning approach similar to AR-PAM. By using large annular shaped transducers, we achieve high spatial resolution in all dimensions also for large imaging depths at lower costs compared to PACT. In contrast to AR-PAM, an array of such annular detectors in conjunction with a delay-and-sum-technique provides relatively constant lateral resolution over a large range of imaging depth. Hence, PASMac may find clinical application e.g. in early breast cancer detection. Passler et al. demonstrated such large depth of field imaging (DOF) with a ring-shaped piezoelectric transducer [13]. It was shown that a ring-shaped transducer focusses on the symmetry axis and provides a large depth of field. However, images acquired by a single annular detector often suffer from X-shaped artifacts, arising when a small point-like structure is imaged. To suppress these aberrations, arrays of ring-shaped transducers with varying diameters can be used. Annular detector arrays for photoacoustic microscopy were, e.g., presented in [14], [15], [16], [17]. A piezoelectric annular detector array for large DOF photoacoustic imaging was demonstrated by Passler et al. [18]. We pursue a similar approach in this work. However, instead of piezoelectric transducers, we detect the ultrasonic waves with fiber-optic ring-shaped detectors. Using optical fibers rather than piezoelectric films offers several advantages: They are transparent for excitation light, they do not require electrical shielding and have low cross-talk, their sensitivity is independent on the incident angle of the ultrasonic wave, their bandwidth is practically only limited by the used photodetector – maybe the most important advantage – they offer a higher sensitivity.

The structure of the paper is as follows. After discussing the system and its components in Section 2, we present signal processing, image formation, sample preparation, and details on the performed experiments and simulations in Section 3. In Section 4, the results of the conducted experiments are presented. It follows a discussion of the results of the experiments, possible enhancements of the system, and its potential for photoacoustic imaging.

## 2 System design

### 2.1 System overview

**Fig. 1:**
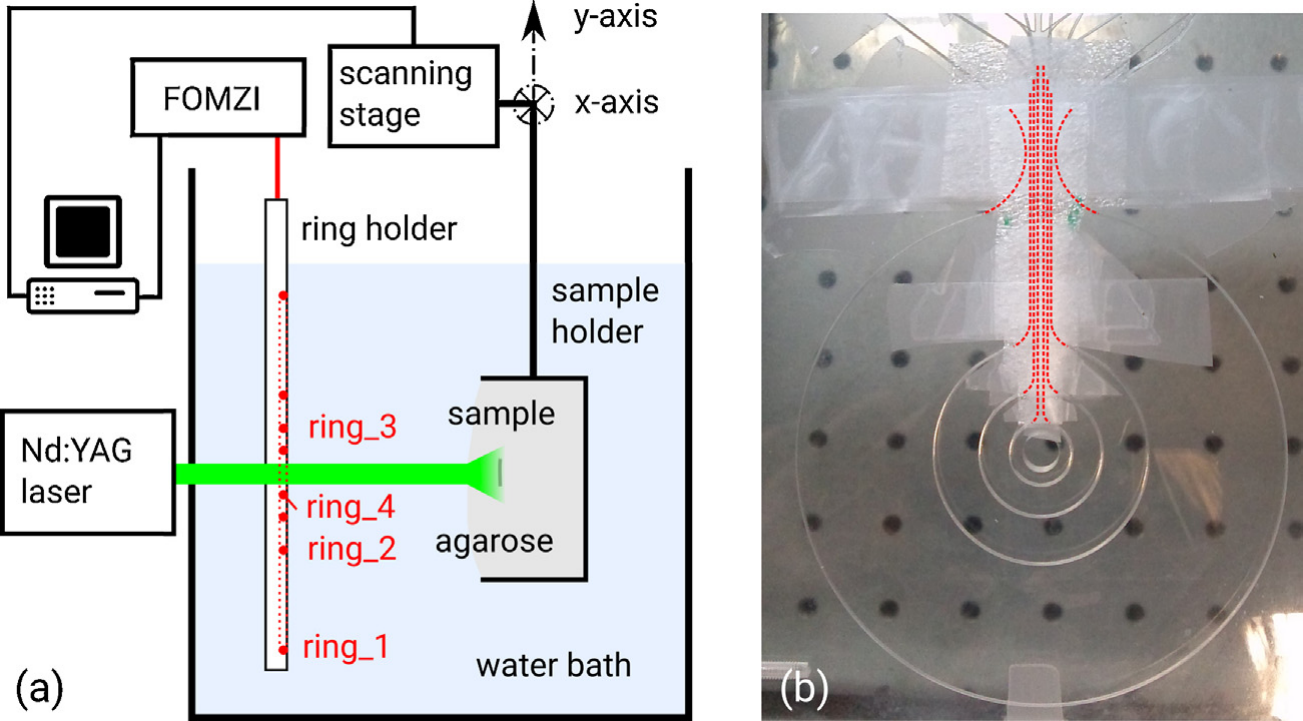
(a) Schematic of the setup. A sample is embedded in agarose and moved parallel to the ring-plane by a scanning stage. Photoacoustic waves within the sample are excited by pulses from a frequency doubled Nd:YAG laser. The photoacoustic signals are picked up by graded-index polymer optical fiber rings which are part of fiber-optic Mach–Zehnder interferometers (FOMZI). (b) A photograph of the acrylic glass fiber-holder. The overlaid dotted red lines indicate the path of the fibers where they are not visible in the photograph. The fibers are held in place by ring-shaped notches. These ring-notches cannot be closed to form a complete circle as the optic fibers have to be fed into the notches with a minimal bend radius of 5 mm. The rounded openings (at 12 o’clock in the picture) ensure proper feeding of the fibers. Bubble wrap insulates the fibers from ultrasound where they deviate from the ring-shape. Duct tape fixes the bubble wrap and the fibers in the feeding lines. (For interpretation of the references to color in this figure legend, the reader is referred to the web version of this article.)

The schematic of the setup is shown in Fig. 1a. A frequency doubled Nd:YAG laser (Continuum Surelite, 20 Hz repetition rate, 6 ns pulse duration, 532 nm center wavelength) is used to generate photoacoustic signals within the samples. The annular ultrasound detectors are made of graded-index polymer optical fibers (GIPOFs). Circular notches in a polymethyl methacrylate (PMMA) plate maintain the annular shape of the GIPOFs (see photograph in Fig. 1b). The ring diameters are chosen to obtain a flexible sensor with a large depth of field. In general, larger rings provide a higher numerical aperture (NA) than smaller rings and can, thus, provide a higher lateral resolution also in larger imaging depths. However, the size of the largest ring is practically limited in order to allow reasonable handling of the sensor, whereas the diameter of the smallest ring is limited by the minimal allowed bending radius of the fiber of 5 mm. Artifact-suppression benefits from substantial different ring dimensions [13] and hence we have chosen the ring diameters to be 80 mm (denoted ring_1), 35 mm (ring_2), 20 mm (ring_3), and 10 mm (ring_4). Using PMMA for the ring holder offers two advantages: (i) it is transparent for light within the frequency range used for photoacoustic excitation and therefore does not restrict sample illumination and (ii) it is acoustically well matched to the fibers and to water, which reduces imaging artifacts caused by reflections. A circular opening in the center of the fiber holder offers the opportunity to simultaneously measure the ultrasonic waves at the ring's joint axis with a reference transducer, e.g., a needle hydrophone. Samples are embedded in agarose and position-scanned in a plane perpendicular to the ring axes by a two-dimensional scanning stage (Märzhäuser Scan IM 120 × 100). The GIPOFs are part of fiber-optic interferometers. The interferometers and the scanning stage are controlled by an image acquisition software running on a PC.

### 2.2 Ultrasound detection based on fiber-optic Mach–Zehnder interferometer (FOMZI)

**Fig. 2:**
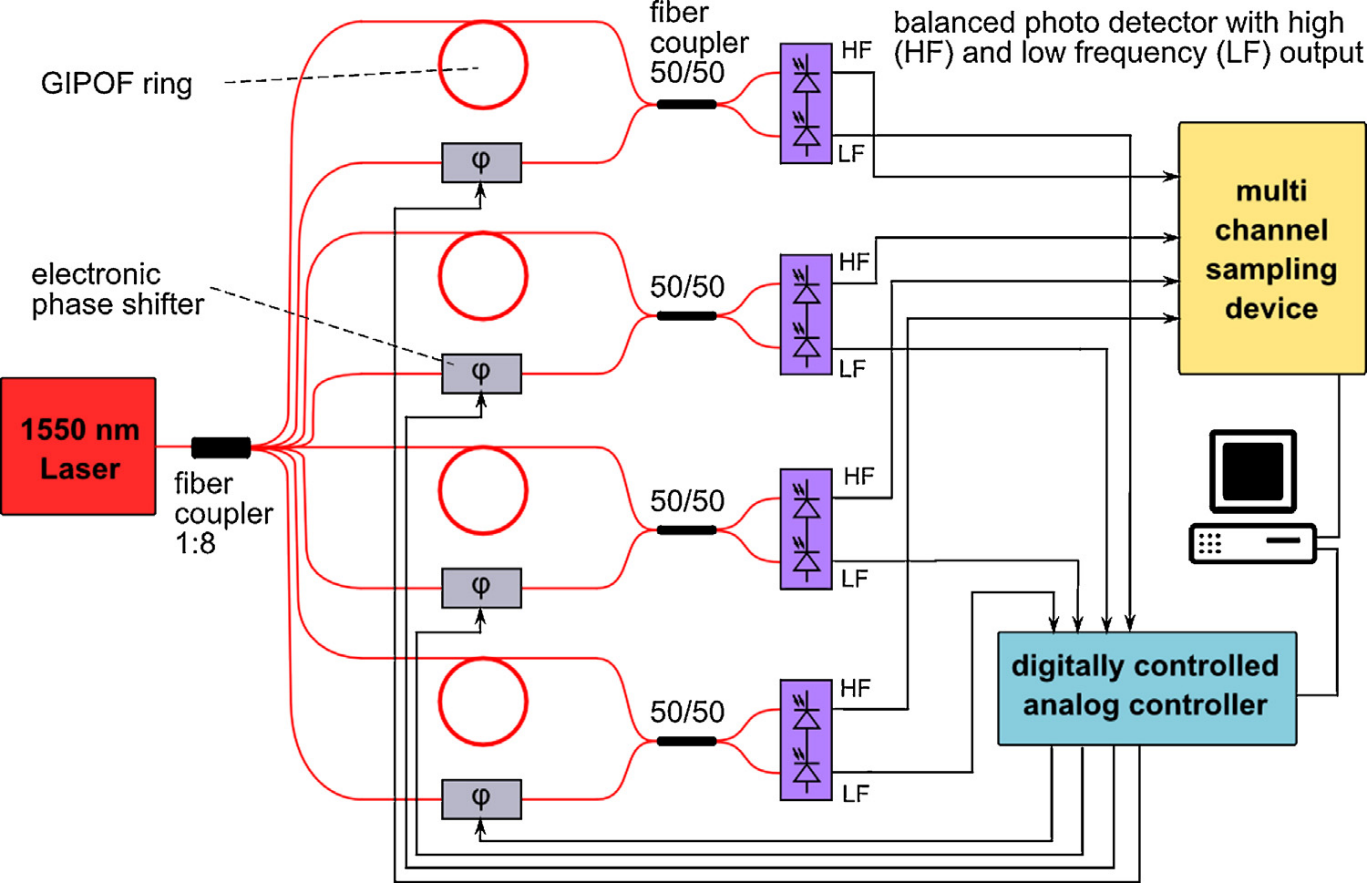
Schematic of the annular ultrasound detector array based on fiber-optic Mach–Zehnder interferometers. A 1550 nm cw laser is connected to an 1:8 fiber coupler. Four outlets of the coupler are connected to the sensing fiber-rings. The remaining outlets supply the reference paths of each interferometer. Electronic phase shifters (*φ*) stabilize the working point. The superposed light of the sensing and the reference arms is converted into electric signals with balanced photodetectors. The bandwidth of the low-frequency output of these detectors is limited by the desired minimal ultrasound frequency. The LF signal drives the electronic phase shifters via analog controllers. The ultrasonic signals (HF) are sampled with a multichannel sampling device.

Ultrasound detection with fiber-optic interferometers relies on the strain-optical effect. Strains within a fiber, induced by ultrasonic waves, change the refractive index in the fiber core [19]. The resulting phase shift of light propagating in the fiber core can then be detected by interferometric means. Fig. 2 shows the schematic of the used four channel detector array. A fiber laser (Koheras AdjustiK) with a maximum output power of 200 mW and low phase noise supplies the four fiber-optic interferometers. Laser light with a wavelength of 1550 nm is distributed into a sensing arm and a reference arm for each of the four channels using an 1:8 fiber coupler. The sensing arms consist of graded-index polymer optical fibers (Chromis Fiberoptics, 57 μm core diameter), which are shaped into rings. In comparison to standard glass optical fibers the GIPOFs offer higher phase shifts and a more flat frequency response [19]. An electronic phase shifter (Phoenix Photonics) is used to establish the working point at a phase-difference between reference and measurement arms of *π*/2 (for a detailed discussion see Section 2.4). At the working point, the change in intensity of the superposed light due to a change of the phase difference is maximal. Superposition of the light in the reference and sensing arms is achieved in a 50/50 fiber coupler. A self-made balanced photodetector (see Section 2.3) transforms the light into high-frequency (HF) and low-frequency (LF) electric signals. These outputs provide the signals for ultrasound detection and stabilization of the working point, respectively. Balanced detection is applied in order to suppress common-mode disturbances, e.g. intensity noise of the laser. The output of the fiber laser is chosen in a way that both photodiodes operate just below saturation. Thereby, twice the output power can be used compared to using non-balanced detection employing only one photodiode. This leads to a doubling of the ultrasonic sensitivity. As the quantum noise is also increasing with laser power, the increase in the signal-to-noise ratio is $\sqrt{2}$. The HF output is proportional to the photoacoustic signal and is recorded with a multichannel sampling device (National Instruments PXI-5105, 12 bit, 60 MHz bandwidth) and a personal computer (PC). The LF output represents low-frequency disturbances of the phase difference (such as spurious drift) and is fed to an analog controller which drives the electronic phase shifters. A software, running on the PC, is used to configure the controllers via an USB connection. By design, the dominant contribution to the signal noise originates from the quantum noise of the laser light.

### 2.3 Balanced photodetector

**Fig. 3:**
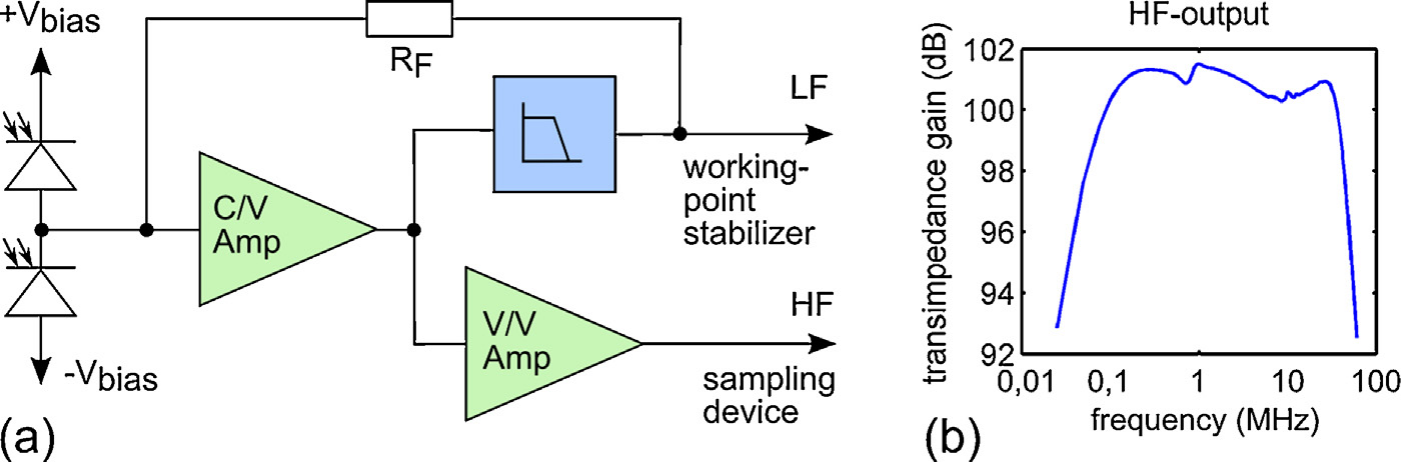
(a) Schematic of the in-house designed balanced photodetector. Two reversed-biased photodiodes transform the output of the interferometer into a photocurrent. This current is fed to a transimpedance converter (C/V). A voltage amplifier elevates the output of the C/V stage to a net of 101 dB. A feedback loop, consisting of an active low-pass filter and a resistor (R_F_), prevents the detector from saturation. A tap of the feedback loop provides the LF-signal, required for the stabilization of the working point. (b) HF-gain measured from 20 kHz to 100 MHz. The −3 dB-points are 54 kHz and 43 MHz.

Based on our experience we decided to build a photodetector with a transimpedance-gain of about 100 dB. This gain is required to transform the output of the photodiodes at typically occurring phase shifts to a voltage which can be measured by a digital sampling device. For the detection bandwidth we chose an upper cut-off frequency of 40 MHz. The photoacoustic signals may be superimposed with low-frequency disturbances caused, for example, by low-frequency sonic waves or thermal drifts. These interferences can be much stronger than the actual photoacoustic signals and could result in saturation of the photodetector. In order to prevent this, the gain of the photodetector has to fall off steeply for low frequencies (<100 kHz). Low-frequency disturbances, however, also affect the working point of the interferometer. Thus, the detector has to supply the analog controller with this low-frequency components of the phase difference. These requirements resulted in an in-house development of a balanced photodetector with high- and low-frequency outputs based on a low-noise high-gain operational amplifier (Texas Instruments, OPA2846). Fig. 3a illustrates its schematic. The differential photocurrent of two serial gallium-arsenide PIN photodiodes (JDSU, ETX100) is transformed into a voltage by a transimpedance amplifier (C/V). In order to achieve stable operation, high bandwidth, and high gain, the input capacitance of C/V-converter has to be minimized. This is accomplished in two ways: First, by reverse-biasing of the photodiodes and, second, by using an active low-pass filter feedback loop instead of a bandpass filter for splitting the signal into its HF and LF components. The feedback loop is tapped and provides the LF-signal. A voltage amplifier (V/V) amplifies the output of the C/V-converter to a total gain of >100 dB.

The detector was tested with an amplitude modulated continuous wave laser diode by sweeping the modulation frequency in the range of 20 kHz to 100 MHz [20]. The resulting transimpedance gain versus frequency is plotted in Fig. 3b. The photodetector achieves a gain of 101 dB between the 3 dB points of 54 kHz and 43 MHz. If the HF-output is terminated with 50 Ohms, the root-mean-square of the electrical noise is measured as 4.2 mV and the maximal output amplitude is 2 V. Thus, the best possible SNR computes to 53.6 dB.

### 2.4 Stabilization of the working point of the FOMZI

To ensure maximum sensitivity, the working point of the interferometer has to be maintained. At the working point, the phase difference between measurement and reference arm is *π*/2 and the intensity at both photodiodes is equal. Consequently, the voltage at the LF output of the photodetector is zero. Higher frequencies, originating from the photoacoustic signals, are by design suppressed by the low-pass filter and, thus, a controller has to maintain the voltage at the LF output to be zero in order to stabilize the working point. A basic analog two-point controller scheme in conjunction with an electronic phase shifter turned out as a cost-effective solution for stabilization of multichannel fiber-optic Mach–Zehnder interferometers (FOMZIs). Thermal drifts can cause phase deviations higher than the maximal possible shift of the electronic phase shifter, i.e. higher than 50 *π* rad. In this case the controller would become stuck at one of the two output values until the phase shift autonomously reaches *π*/2. During that time, the phase of the FOMZI would run freely and the point of highest sensitivity would not be maintained. Since this is unacceptable in practical applications, we introduced a microcontroller based watchdog which resets the controller when the stabilization of the working point fails for a certain period. In that case, the watchdog additionally causes the image acquisition software (running on a PC) to repeat the measurement at the current position. The controller parameters (output values, timeouts, and reset duration) can be configured by the data acquisition software running on the PC.

## 3 Methods

### 3.1 Phantoms

First, cylinders consisting of a mixture of distilled water, agarose, and Intralipid were fabricated. Intralipid was added to increase optical scattering. The mass fractions of agarose and Intralipid were 2% and 0.5%, respectively. The cylinders had a diameter of 45 mm and heights between 15 mm and 25 mm. The sample objects were then placed on the surface, close to the center of the cylinder. A few drops of agarose/Intralipid mixture were used to fixate the objects on the cylinder. Afterwards, the objects were covered by another cylindrical layer of the agarose/Intralipid mixture with a thickness of approximately 10 mm and a diameter of 45 mm. Three different phantoms were produced: Sample A contained a Rhodamine B dyed polyethylene microsphere (Cospheric, Santa Barbara, CA) with a diameter of about 200 μm. An approximately 5 mm long piece of a pig bristle with a diameter in the range of 120–145 μm was placed in sample B. Sample C contained a 5 mm by 7 mm sized piece of an ink-stained leaf skeleton. The thickness of the leaf vessels vary from 30 μm to 190 μm.

### 3.2 Experiments

#### 3.2.1 Determination of the sensitivity

Sample A was used to determine the sensitivity of the ring detector. Here, the microsphere was positioned at the ring axis at a distance of 90 mm to the ring plane. Additionally to the acquisition with the ring detector array, the pressure at the center of the rings was measured with a calibrated needle hydrophone (Onda HNC1000).

#### 3.2.2 Depth of field

In order to investigate the depth of field of the annular detector array, sample B was imaged at different depths. Measurements were performed at distances of 14.5 mm, 25 mm, 35 mm, 45 mm, 79 mm, 89 mm, and 110 mm. The radiant exposure of the illumination was 10 mJ/cm^2^. The step size of the scan was 40 μm for both the *x*- and *y*-directions and 8-times averaging was performed at each scan-position. One measurement took about 35 min.

#### 3.2.3 Imaging

Sample C was used in a third experiment to test the imaging system on biological structures. The step size of the scan was 40 μm for both the *x*- and *y*-directions, 8-times averaging was performed at each scan-position and the measurement took 6 h. The distance of the leaf skeleton to the detector plane was 30 mm. The radiant exposure was 14 mJ/cm^2^.

### 3.3 Signal processing and image formation

The measured A-scans are normalized according to the individual ultrasound sensitivity of the rings. Subsequently, the pressure signals are Hilbert-transformed and the individual time-of-flight measurements are interpolated to a common *z*-axis [18]. Finally, the contributions of the signals of all rings are summed up. To reduce imaging artifacts originating from the off-axis-sensitivity, coherence weighting is used [18], [17]. By scanning the sample, the obtained A-scans can be arranged to form two-dimensional or three-dimensional images.

### 3.4 Simulations

For simulating the theoretical response of a ring detector we define detection points lying on a circle with radius *R*_*r*_ at a *z*-position of 0

(1) $${\text{r}}_{d}(\varphi )=[R_{r}\cdot \sin(\varphi ),R_{r}\cdot \cos(\varphi ),0],$$

where *φ* is the angle. We further consider a spherical shaped absorber, excited with an infinitely short laser pulse. We can find the pressure *p* measured on any detection point **r**_*d*_ at a distance of Δ*r* from the center of the source **r**_*s*_ by [19]

(2) $$p({\text{r}}_{d},t)=\frac{a}{2}\frac{\Delta r-\nu \cdot t}{\Delta r}\Theta (R_{s}-|\Delta r-\nu \cdot t|),$$

where $\Delta r=\left|{\text{r}}_{d}-{\text{r}}_{s}\right|$, *ν* is the sound velocity, *t* is the time, *R*_*s*_ is the radius of the sphere, *a* describes the amplitude of the wave, and Θ denotes the Heaviside function. The response of the ring detector can then be found by integration over Eq. (2)

(3) $$p(t)=\int_{0}^{2\pi }p({\text{r}}_{d}(\varphi ))d\varphi .$$

Eq. (3) is solved by discretization of Eq. (2) and numerical summation over 101 values of *φ*. To account for the finite bandwidth of the photodetector, the calculated response is filtered by a low-pass filter with a cut-off frequency of 43 MHz. From Eqs. (2), (3), the focusing behavior of an annular shaped detector can be seen. If the pressure source is located on the symmetry axis of the ring, the distance between the source and all points on the ring is equal. This results in a constructive integration of the signal. If the source is off-axis, signals stemming from the absorber arrive at different parts of the ring with varying time delays and therefore partly cancel out by the integration. For more complex shapes than a sphere, we consider the object to be composed of dense lying spherical sources and again apply Eq. (3) for calculating the response.

## 4 Results

### 4.1 Ultrasound sensitivity of the ring detector

**Fig. 4:**
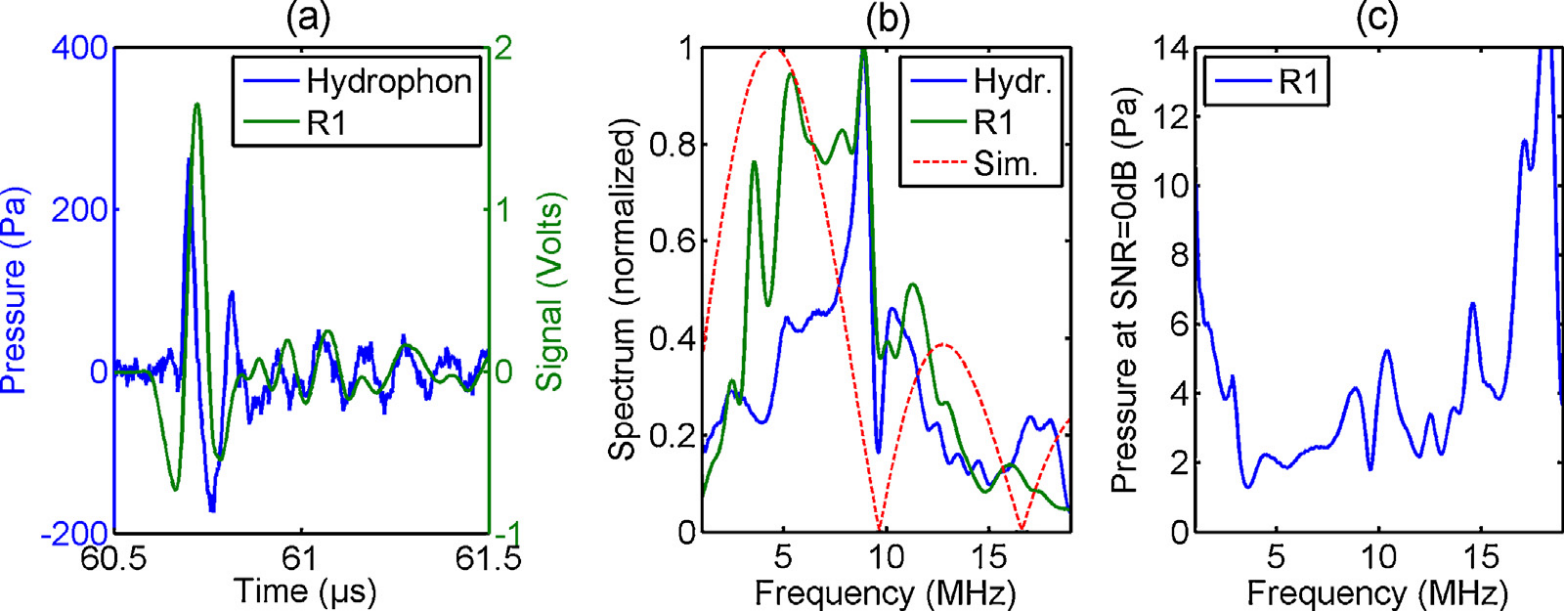
(a) Photoacoustic pressure generated by a Rhodamine microsphere. The pressure was measured at a distance of 9 cm by a hydrophone with 128-times averaging (blue curve). The resulting FOMZI signal of ring_1 (R1, green curve, 8-times averaging) looks similar except for an additional negative peak before the main peak (b). The corresponding spectrum of ring_1 shows higher values at low-frequencies compared to the hydrophone. The steep drop of the spectrum at 10 MHz can also been seen in the simulated spectrum of a sphere with a diameter of 220 μm. (c) Spectral analysis of the ultrasonic pressure at which the response of the sensor equals the rms-noise-level (SNRZ). The average SNRZ is 3.2 Pa in the frequency range from 2 MHz to 16 MHz. (For interpretation of the references to color in this figure legend, the reader is referred to the web version of this article.)

The acoustic pressure measured with the calibrated needle hydrophone is shown as the blue curve in Fig. 4a. The signal was averaged 128-times and exhibits a peak pressure of 262 Pa. The simultaneously acquired response of ring_1 is presented by the green curve in Fig. 4a and shows a peak amplitude of 1.65 V. In this case 8-times averaging was applied. The normalized frequency spectra of the measured signals are presented in Fig. 4b, together with the theoretical pressure-spectrum of a microsphere with a diameter of 220 μm (red curve). The spectral pressure-sensitivity of the ring can be calculated by dividing the spectrum of the ring-signal by the spectrum of the hydrophone-signal. Multiplying this spectral sensitivity with the root-mean-square noise of the ring-detector yields the pressure at which the signal-to-noise ratio (SNR) becomes 0 dB. We denote this value SNRZ. Fig. 4c shows the resulting SNRZ-values in the frequency range from 1 MHz to 19 MHz. The average SNRZ is 3.2 Pa in the frequency range from 2 MHz to 16 MHz.

### 4.2 Depth of field of the annular detector array

**Fig. 5:**
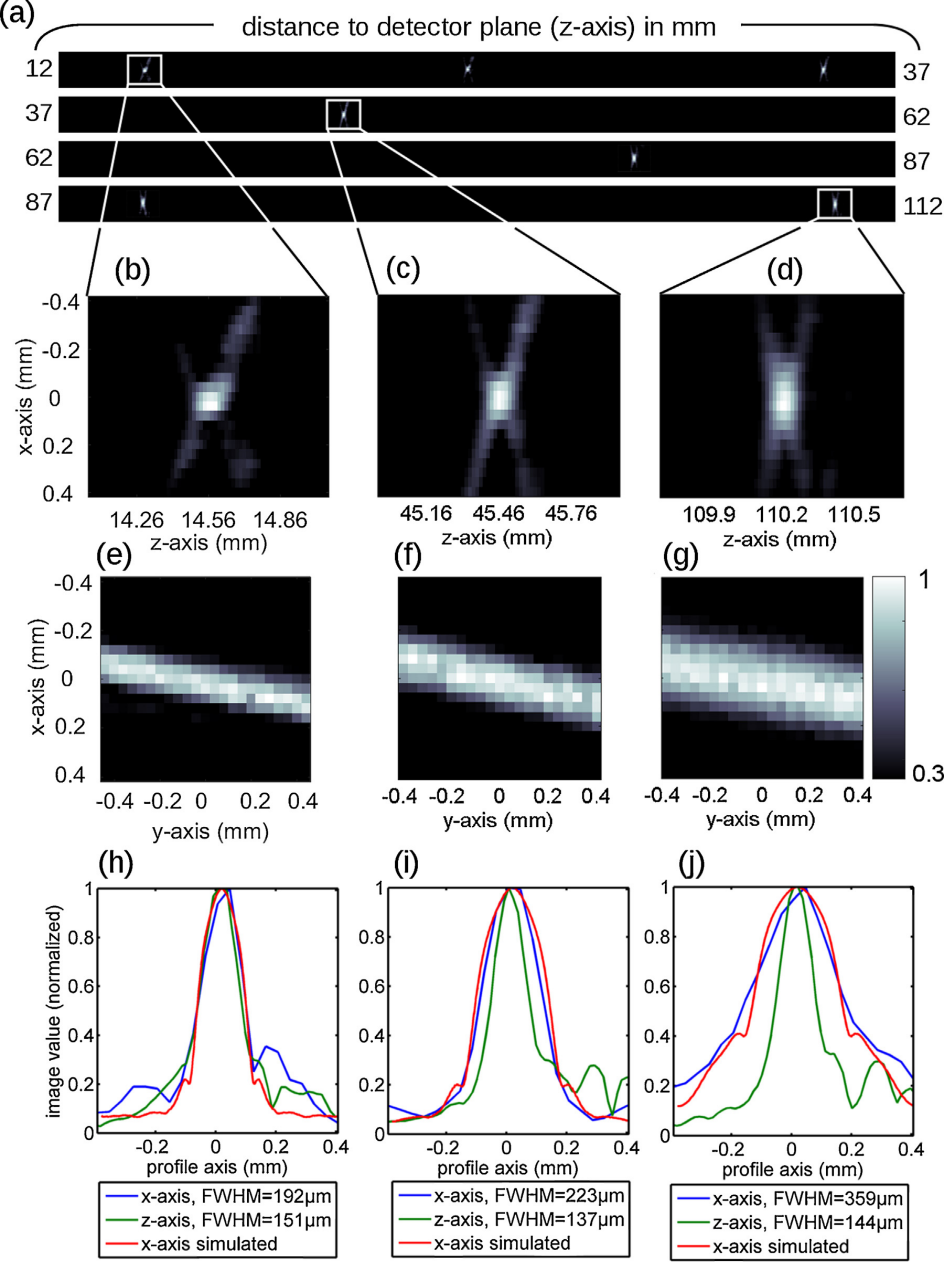
PA images of a black porcine bristle measured at different distances to the detector plane. All pictures are normalized to a maximal numerical value of 1. The colorbar in (g) applies to all cross-sections. (a) *z*–*x*-cross-sections over the entire depth of field. X-shaped imaging artifacts can be identified in the zoomed-in *z*–*x*-cross-sections (b–d). The *y*–*x*-plots (e–g) clearly show the bristle in longitudinal direction without imaging artifacts above a numerical value of 0.3. (h–j) Profiles along the *x*- and *z*-axis. The full-width-at-half-maximum of the *z*-profiles remains constant over depth. The lateral resolution deteriorates with depth. The simulated profiles along the *x*-axis agree well with the measurements.

Fig. 5 shows the resulting cross-sections of the second experiment. In the axial direction, one pixel corresponds to 15 μm. In Fig. 5a the cross-sections in the *z*–*x* plane over the whole depth field are presented. Zoom-ins at *z*-positions of 14.5 mm, 45 mm, and 110 mm are shown in Fig. 5b–d, respectively. X-shaped artifacts can be clearly identified. By using more rings with diameters matched to a distinct imaging depth these artifacts could be reduced significantly. The corresponding cross-sections in the *x*–*y* plane are presented below in Fig. 5e–g. Here, the imaging artifacts are quite low. Fig. 5h–j, shows profiles along the center of the bristle in *x*-direction (blue) and *z*-direction (green). The axial resolution remains constant for all distances and the full-width-at-half-maximum (FWHM) corresponds well to the nominal diameter of the bristle. The lateral resolution degrades with imaging depth and the bristle appears broadened for larger imaging depths. However, the increase in FWHM of the bristle-diameter is below 20% between 15 mm and 45 mm. For imaging depths larger than 10 cm the lateral resolution is still better than 350 μm. For comparison, we present lateral profiles obtained by simulating the response of a cylindrical source with a diameter of 120 μm and a length of 4 mm as the red curve in Fig. 5h–j. The simulated profiles agree well with the measurements. We note that if one would increase the thickness of the agarose, the lateral and axial resolutions would most likely degrade caused by acoustic attenuation. When assuming an attenuation of 1 dB/cm at 1 MHz with a frequency dependence of frequency^1.1^, then the influence of tissue can be estimated by low-pass filtering the photoacoustic signals. E.g. using the data of the measurements at a distance of 4.5 cm (Fig. 5c, f, and i) and simulating additional tissue featuring a thickness of 1 cm above the sample (yielding a total thickness of 2 cm) results in a lateral FWHM of 350 μm and an axial FWHM of 200 μm.

### 4.3 Imaging of a leaf phantom

**Fig. 6:**
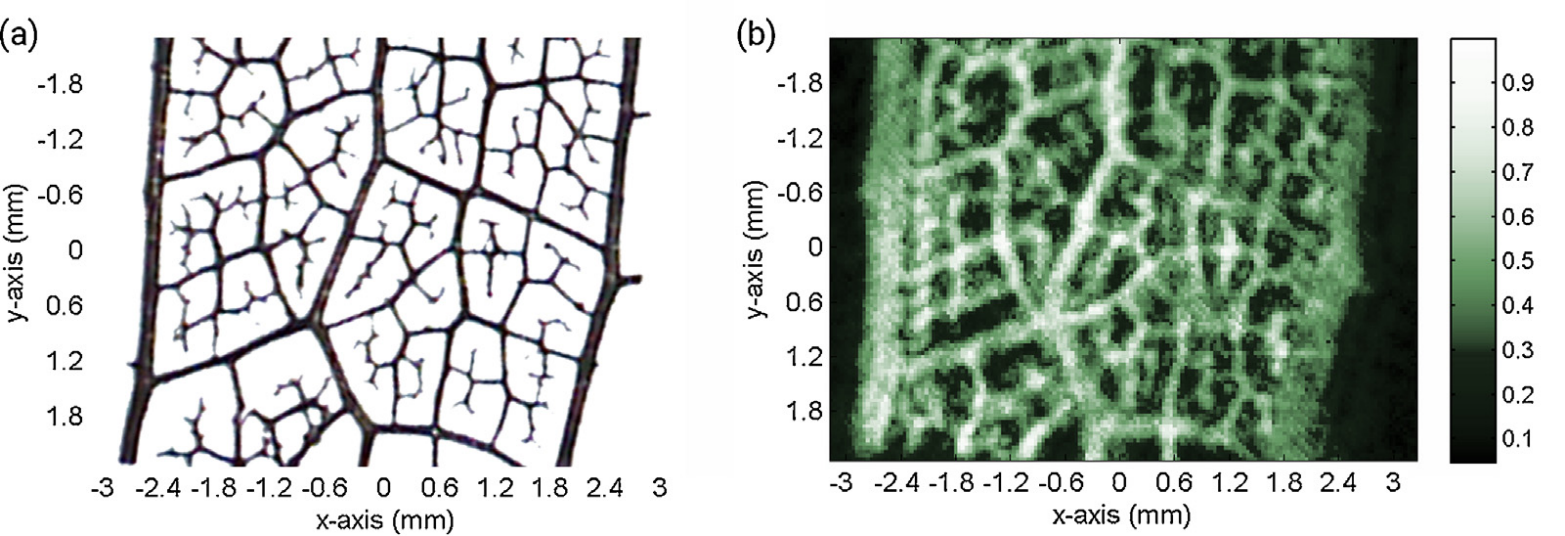
Photography of an ink-stained leaf skeleton before embedding in agarose (a) and corresponding photoacoustic image (b). The strong branches at the left and right boarder of the skeleton measure approximately 160–190 μm in diameter. The photoacoustic image reproduces most of the features of the skeleton. Due to the estimated resolution of 150–200 μm the branches appear clearly broadened. The X-shaped imaging artifacts hardly affect the image quality.

A photography of the sample C before embedding is shown in Fig. 6a. A cross-section of the acquired photoacoustic data is shown in Fig. 6b. The smallest structures which are observable in the photoacoustic image stem from 30 μm thick vessels. The lateral resolution was estimated to be 150–200 μm, leading to a broadening of these structures. Besides the broadening, the skeleton is replicated properly. During sample preparation, some of the small features were bent out of the leaf-plane. Due to the high axial resolution of the photoacoustic image, these features are not visible in the cross-section parallel to the leaf-plane.

## 5 Discussion

In the current implementation of PASMac, the imaging speed is too slow for most biomedical applications. The slow imaging speed is mainly determined by the scanning stage and the scanning process. At each individual scan position the stage is halted and the measurement is performed only after a settling time of 0.2 s. This delay is necessary to reduce vibrations of the scanning stage. In order to speed-up image acquisition, we plan to use a different scanning stage together with a different scanning mode, scanning a whole line without stopping at each measurement position. In such a scenario, the stage will move at a constant speed while in the scanning area. This results in a fixed relation between measurement time, i.e. time of the excitation laser pulse, and the position of the stage. Alternatively, the stage could be programmed to send trigger pulses to the laser at distinct distances. In both cases, by synchronizing the laser pulse with the position of the stage, individual measurements can be correlated to a distinct position. For a laser repetition rate of 20 Hz we expect the required time for a single line with 100 points to be slightly above 5 s. By using an excitation laser with higher repetition rate, obviously, this time could be lower.

In the first experiment, we measured a microsphere with a diameter of about 220 μm and compared the signals and spectra obtained by a needle hydrophone and a ring transducer. The obtained time traces look similar, however, also small differences like an additional negative peak in the signal obtained by the ring can be found. Both obtained time traces differ from the ideal N-shaped pulse send out by a spherical source. The differences can also be seen in the respective frequency spectra, which differ from the theoretical frequency response of a microsphere. We attribute these differences to two effects: First, neither the ring transducer nor the needle hydrophone exhibit an ideal frequency response. The differences in frequency response consequently lead to differences in the time traces. Second, the ideal N-shaped pressure pulse is only observed if the sphere is illuminated homogeneously, i.e. if the energy density is equal at every point within the sphere. In our case, the sphere is illuminated in backward mode from the same side as the transducers are placed. Thus, more light is absorbed at the part of the shell pointing towards the transducers than at the backside of the sphere. This leads to an ultrasonic wave which differs from the ideal form and exhibits higher directionality than a spherical wave. Caused by the higher directionality, the shape of the pressure wave obtained by the needle hydrophone could be slightly different than the waveform obtained by the ring. Also, the pressure amplitudes at the hydrophone could be higher than at the ring. In this case, we underestimated the performance of our sensor and the average SNRZ could be lower than the calculated 3.2 Pa.

The imaging resolution of the system can be estimated by the full-width-at-half-maxima (FWHM) determined in the second experiment. Due to the finite size of the bristle, the actual resolution of the sensor should be better. In thick biological samples acoustic attenuation will lead to deterioration of the resolution. Hence, the resolution estimated from the bristle-measurements might not be reached. In this case, the resolution will be limited by the acoustic attenuation rather than by the performance of the presented sensor. In the second experiment we compared the measured FWHM to simulations and found an excellent agreement of the obtained values. The simulation procedure was also used to simulate the sensor-response to the ultrasound signals stemming from a small microsphere with a radius of 5 μm. We found that the simulated lateral resolution matches well with the theoretical resolution for spherical focusing ultrasound transducers which is given by $0.71\cdot \nu /\left(\text{NA}\cdot f\right)$. Here, *f* is the frequency of the ultrasonic wave, *ν* corresponds to the sound velocity, and NA denotes the numerical aperture. For imaging depths below 50 mm, we found that the formula underestimates the resolution by about 5% in respect to the simulation. For an imaging depth of 110 mm, the discrepancies were below 20%. Thus, the formula derived for the resolution of spherical focusing ultrasound transducers might be a good starting point for the design of future fiber-optic ring-arrays.

From the second and third experiments we found, that the image quality could be improved by excluding data of individual rings. Specifically, for shorter imaging depths ring_1 and for larger depths ring_4 were not considered. We identified various reasons for this behavior. As discussed above, unidirectional illumination of the samples could lead to a directionality of the photoacoustic signals. If the generated photoacoustic signals are directional, i.e. if they deviate from the ideal spherical form, then the outer rings may receive less signal compared to the inner rings. With decreasing imaging depths this effect is intensified. We expect this effect to decrease, when the sample is illuminated from various directions instead. Another problem originates from the ring-shaped notches in the fiber-holder. Ultrasonic waves approaching the ring at shallow angles in respect to the ring-plane cannot reach the fiber directly because they are partly reflected and refracted by the PMMA fiber-holder. This issue could be addressed by using a different notch-profile, e.g. with sloped walls. Both issues lead to a decreased sensitivity of the larger rings with decreasing imaging depth. On the other hand, for larger imaging depths the lateral focus of the inner rings is significantly larger than for the outer rings, thereby reducing the resolution of the image. It is therefore important to match the ring-diameters to the desired imaging depth. In this paper, we demonstrated imaging in the range between 14.5 mm and 110 mm, requiring a large difference in the ring diameters. In future we will focus to a practically more relevant depth region below 50 mm. AR-PAM in the targeted imaging depth was, e.g., shown by Song et al. [21]. Here, a lateral resolution of 560 μm at a depth of 19 mm was demonstrated. In Section 4.2 we estimated the lateral resolution of our setup for the same thickness of tissue to be about 350 μm. Reducing the maximum imaging depth to 50 mm will also result in a smaller and less bulky sensor. We expect the diameter of the largest ring of such a sensor-array to be about 40–50 mm. This size corresponds to the active aperture of many common ultrasonic line array transducers. Thus, we suppose that this size should be no limitation for various applications.

In [22] we demonstrated the feasibility to read-out the signals from 16 fiber-optic detection channels in parallel. We are planning to extend our detector array to at least 8 rings, whereby the ring diameters should be optimized for imaging depths between 5 mm and 50 mm. Using 8 rings with appropriate diameters should reduce imaging artifacts further and should lead to an enhanced SNR. In addition, the bandwidth of the photodetector should be matched to the targeted imaging depth. If acoustic attenuation in tissue [23], [24], [25] is considered a photodetector with less bandwidth should perform better. For an imaging depth of 5–50 mm we will limit the bandwidth to 10 MHz instead of the currently used 43 MHz. Thus, we expect the SNR to improve by a factor of 2.

An enhanced version of the current PASMac system has the potential to fill a gap between existing AR-PAM and array-based PACT systems. PASMac offers higher imaging depths than AR-PAM with resolutions similar or even better than PACT. In contrast to many PACT implementations, PASMac operates in backward mode and has therefore lower requirements on sample-accessibility. Additionally, since the detector is transparent, there are no restrictions on the illumination-scheme. The large annular fiber-optic interferometers of PASMac allow a high sensitivity in the range of a few Pascals at a bandwidth >15 MHz. However, since the method includes the scanning of a large detector, acquisition-times will be too long for real-time three-dimensional volumetric imaging. We therefore target on the acquisition of photoacoustic B-scans. With a laser repetition rate of 100 Hz, a B-scan consisting of 100 A-scan could be acquired in about 1 s.

When developing a new method, also the expected costs of such a system should be assessed. Expenses for the detection-laser and for the stabilization of the working point are noteworthy. However, since rather low-cost equipment from telecommunication [26] can be used, the investment for optical detection remains affordable. In comparison to PACT-systems with a large number of channels [7], [10], [12], we think that the costs of the proposed PASMac-system are significantly lower. PACT has shown high potential in various biological and medical areas [3]. For example, in early breast cancer detection and cancer monitoring PACT methods are currently under development and entering clinical trials [8], [10], [27], [28]. Here, PASMac has the potential to provide images with higher sensitivity and fewer costs in special cases. In the future, we intend to investigate the applicability of using such a device for breast imaging. In contrast to full breast or large field of view tomography devices our aim is the detailed imaging of defined regions of interest (ROI) that previously have been defined by other imaging methods. In addition to the diagnosis of neovasculature, such a tool could be useful for monitoring the treatment of an already diagnosed tumor.

## 6 Conclusion

We introduced a system for large depth of field photoacoustic scanning macroscopy (PASMac). For ultrasound detection, a fiber-optic annular transducer array was used. The ring-shaped sensing fibers consist of graded-index polymer optical fibers, which offer higher sensitivity than glass optical fibers. Refractive index changes in the optical fibers, caused by the impinging ultrasonic waves, were demodulated by interferometric means. We demonstrated imaging in depths ranging from 1.45 cm to 11 cm. For samples covered with 1 cm of agarose, the lateral resolution was determined to be below 200 μm at a depth of 1.5 cm and below 230 μm at a depth of 4.5 cm. For an imaging depth of 11 cm the lateral resolution was still better than 360 μm. By comparing signals from a calibrated hydrophone and the fiber-optic ring detector, we calculated the minimum detectable pressure to be in the order of 3 Pa for a detection bandwidth of 43 MHz. Additionally, we demonstrated photoacoustic macroscopy on a leaf skeleton phantom.

## Conflicts of interest

The authors declare that there are no conflicts of interest.
